# Chaste: Cancer, Heart and Soft Tissue Environment

**DOI:** 10.21105/joss.01848

**Published:** 2020-03-13

**Authors:** Fergus R Cooper, Ruth E Baker, Miguel O Bernabeu, Rafel Bordas, Louise Bowler, Alfonso Bueno-Orovio, Helen M Byrne, Valentina Carapella, Louie Cardone-Noott, Cooper Jonatha, Sara Dutta, Benjamin D Evans, Alexander G Fletcher, James A Grogan, Wenxian Guo, Daniel G Harvey, Maurice Hendrix, David Kay, Jochen Kursawe, Philip K Maini, Beth McMillan, Gary R Mirams, James M Osborne, Pras Pathmanathan, Joe M Pitt-Francis, Martin Robinson, Blanca Rodriguez, Raymond J Spiteri, David J Gavaghan

**Affiliations:** 1 Wolfson Centre for Mathematical Biology, Mathematical Institute, University of Oxford, Oxford, UK; 2 Centre for Medical Informatics, Usher Institute, University of Edinburgh, Edinburgh, United Kingdom; 3 Department of Computer Science, University of Oxford, Oxford, UK; 4 Division of Cardiovascular Medicine, Radcliffe Department of Medicine, University of Oxford, Oxford, UK; 5 Research IT Services, University College London, London, UK; 6 Centre for Biomedical Modelling and Analysis, Living Systems Institute, University of Exeter, Exeter, UK; 7 School of Psychological Science, University of Bristol, Bristol, UK; 8 School of Mathematics & Statistics, University of Sheffield, Sheffield, UK; 9 Bateson Centre, University of Sheffield, Sheffield, UK; 10 Department of Computer Science, University of Saskatchewan, Canada; 11 Centre for Mathematical Medicine & Biology, School of Mathematical Sciences, University of Nottingham, Nottingham, UK; 12 Digital Research Service, University of Nottingham, Nottingham, UK; 13 Faculty of Biology, Medicine and Health, University of Manchester, Manchester, UK; 14 School of Mathematics and Statistics, University of Melbourne, Victoria, Australia; 15 Office of Science and Engineering Laboratories (OSEL), Center for Devices and Radiological Health (CDRH), U.S. Food and Drug Administration (FDA), Silver Spring, MD 20993, USA

## Abstract

Chaste (Cancer, Heart And Soft Tissue Environment) is an open source simulation package for the numerical solution of mathematical models arising in physiology and biology.

To date, Chaste development has been driven primarily by applications that include continuum modelling of cardiac electrophysiology (‘Cardiac Chaste’), discrete cell-based modelling of soft tissues (‘Cell-based Chaste’), and modelling of ventilation in lungs (‘Lung Chaste’). Cardiac Chaste addresses the need for a high-performance, generic, and verified simulation framework for cardiac electrophysiology that is freely available to the scientific community. Cardiac chaste provides a software package capable of realistic heart simulations that is efficient, rigorously tested, and runs on HPC platforms. Cell-based Chaste addresses the need for efficient and verified implementations of cell-based modelling frameworks, providing a set of extensible tools for simulating biological tissues. Computational modelling, along with live imaging techniques, plays an important role in understanding the processes of tissue growth and repair. A wide range of cell-based modelling frameworks have been developed that have each been successfully applied in a range of biological applications. Cell-based Chaste includes implementations of the cellular automaton model, the cellular Potts model, cell-centre models with cell representations as overlapping spheres or Voronoi tessellations, and the vertex model. Lung Chaste addresses the need for a novel, generic and efficient lung modelling software package that is both tested and verified. It aims to couple biophysically-detailed models of airway mechanics with organ-scale ventilation models in a package that is freely available to the scientific community.

Chaste is designed to be modular and extensible, providing libraries for common scientific computing infrastructure such as linear algebra operations, finite element meshes, and ordinary and partial differential equation solvers. This infrastructure is used by libraries for specific applications, such as continuum mechanics, cardiac models, and cell-based models. The software engineering techniques used to develop Chaste are intended to ensure code quality, re-usability and reliability. Primary applications of the software include cardiac and respiratory physiology, cancer and developmental biology

## The software

Chaste is available on GitHub https://github.com/Chaste/Chaste, and the current stable release is version 2019.1. Please see the Readme.md file on the Github repository for links to the Chaste wiki and install guides.

Previous publications about Chaste have detailed the rationale for, and design principles behind, Chaste (Pitt-Francis et al., 2009), as well as the main application areas of Chaste up to 2013 (Mirams et al., 2013).

Chaste places an emphasis on reproducibility and verification and, as such, extensive automated testing is used to ensure software quality and reliability. A series of test suites must all pass before any commit is considered a release-candidate. Most testing is performed on Long Term Support (LTS) versions of Ubuntu Linux, with unit tests additionally being run on macOS.

Testing includes compilation of all libraries with GCC, Clang and Intel C++ compilers; extensive unit testing; performance profiling to identify any slowdowns over time; memory testing with valgrind; verification of code coverage; and running unit tests with different combinations of dependencies to ensure portability. The output of these tests is available at https://chaste.cs.ox.ac.uk/buildbot/.

Since 2013, Chaste has substantially changed to modernise its infrastructure and to enable new science. In terms of infrastructure, Chaste now uses a modern CMake build system, the C++14 language standard, and makes extensive use of BuildBot for continuous integration. In terms of science, Lung Chaste is entirely new and allows the use of Chaste in a new scientific domain. In Cardiac Chaste, we can now create algebraic Jacobians for CellML ODE systems, which can improve speed of simulation for cardiac action potential and tissue simulations (Cooper, Spiteri, & Mirams, 2015), and metadata annotations of CellML files have replaced manual specification of variables in configuration files. Cell-based Chaste has been overhauled to improve flexibility. Changes include hierarchies of simulation modifiers, information writers, cell-cycle models, subcellular reaction network models, and numerical methods that allow new customisation points in almost every area of all cell-based simulations. In addition, simulation output has been standardised to use VTK, a standard and powerful visualisation framework, and some cell-centre simulations now run in parallel using MPI.

## Comparison with other software

Chaste provides substantial common infrastructure enabling a wide range of applications across multiple disciplines. Common elements include meshing, solving differential equations, input/output and continuum mechanics, and these form a platform for Cardiac, Cell-based and Lung Chaste.

A key goal of Chaste is to enable the implementation of many different modelling frameworks. This not only allows a user to select the most appropriate tool for their research but, importantly, enables the comparison of different modelling frameworks to better understand the benefits and drawbacks of each (Osborne, Fletcher, Pitt-Francis, Maini, & Gavaghan, 2017). This is an explicit design goal of Chaste, which focusses on the flexibility of implementing

multiple models rather than (for example) building a graphical user interface. See Table 1 for a comparison of alternatives to Chaste in specific domains, with all other software tools implementing a single modelling framework.

## Installation

Installation of Chaste has been greatly simplified through the development of a Docker image https://github.com/chaste/chaste-docker. Docker is a lightweight, open-source virtualisation technology for running encapsulated applications (‘containers’) on all major operating systems at near-native speed. This enables Chaste (including all dependencies, environment settings, convenience scripts and the latest precompiled release) to be downloaded and installed with just a single command. Isolating Chaste within a container also means that its dependencies and those installed on the user’s host system can coexist without interference or version conflicts.

In addition to simplifying the set-up and execution of Chaste, importantly this also enhances its reproducibility by providing a homogeneous computational environment regardless of the underlying operating system and hardware. Not only is the Chaste source code version-controlled, but so too are the dependencies, configuration settings and environment variables used to build and run it. This means that collaborators and reviewers can easily and consistently reproduce results (to within machine precision) on any platform while developers can seamlessly migrate and scale-up their simulations from a laptop to a workstation or HPC cluster.

## Example usage

Chaste has tutorials to walk users through basic functionality for each application area. Tutorial examples are bundled for each specific release version, and examples for this release are available at https://chaste.cs.ox.ac.uk/chaste/tutorials/release_2019.1.

Tutorials take the form of C++ header files that each define ‘tests’ in the Chaste testing infrastructure. These tests must be compiled and run to produce an output, which can be visualised using ParaView.

In the following sections we showcase a specific tutorial for each of cardiac, cell-based, and lung Chaste, with minimal commands necessary to reproduce the output shown.

## Cardiac example

Here we demonstrate how to run and visualise a three-dimensional monodomain cardiac simulation. This follows the tutorial TestMonodomain3dRabbitHeartTutorial which simulates the result of an electrical stimulus being applied to a realistic rabbit heart geometry. Assuming that

1 Chaste has been installed on Ubuntu Linux (or is running within a Docker container),
2 the Chaste source code exists at $CHASTE_SOURCE_DIR,
3 the environment variable $CHASTE_TEST_OUTPUT is set to a valid directory,

a minimal set of commands to build and run the tutorial is as follows:

mkdir build && cd build

cmake $CHASTE_SOURCE_DIR

make TestMonodomain3dRabbitHeartTutorial

ctest -R TestMonodomain3dRabbitHeartTutorial

This will produce output in the following directory:

$CHASTE_TEST_OUTPUT/Monodomain3dRabbitHeart

To view the results evolving over time as an animation in ParaView it is necessary to postprocess the results with the following command:

cd $CHASTE_TEST_OUTPUT/Monodomain3dRabbitHeart/vtk_output

python $CHASTE_SOURCE_DIR/python/utils/AddVtuTimeAnnotations.py \

results.vtu annotated_results.vtu

To visualise the output, open the file annotated_results.vtu in ParaView, and select to colour by V (voltage).

## Cell-based example

Here we demonstrate how to run and visualise a cell sorting simulation using Chaste’s vertex model implementation. This follows the tutorial TestRunningDifferentialAdhesionSimulationsTutorial. Assuming that

1 Chaste has been installed on Ubuntu Linux (or is running within a Docker container),
2 the Chaste source codeexists at $CHASTE_SOURCE_DIR,
3 the environment variable $CHASTE_TEST_OUTPUT is set to a valid directory,

a minimal set of commands to build and run the tutorial is as follows:

mkdir build && cd build

cmake $CHASTE_SOURCE_DIR

make TestRunningDifferentialAdhesionSimulationsTutorial

ctest -R TestRunningDifferentialAdhesionSimulationsTutorial

This will produce output in the following directory:

$CHASTE_TEST_OUTPUT/TestVertexBasedDifferentialAdhesionSimulation

To visualise the simulation, open the file results.pvd in ParaView, choose to colour by‘Cell types’, and display ‘Surface With Edges’.

## Lung example

Here we demonstrate how to run and visualise the lung airway generation tutorial. This follows the tutorial TestAirwayGenerationTutorial which statistically generates lung airways given initial geometry segmented from a CT scan. Assuming that

1 Chaste has been installed on Ubuntu Linux (or is running within a Docker container),
2 the Chaste source code exists at $CHASTE_SOURCE_DIR,
3 the environment variable $CHASTE_TEST_OUTPUT is set to a valid directory,

a minimal set of commands to build and run the tutorial is as follows:

mkdir build && cd build

cmake $CHASTE_SOURCE_DIR

make TestAirwayGenerationTutorial

ctest -R TestAirwayGenerationTutorial

This will produce output in the following directory:

$CHASTE_TEST_OUTPUT/TestAirwayGenerationTutorial

To visualise the generated airway geometry, open the file example_complete_conducting_ airway.vtu in ParaView. Application of an ‘Extract Surface’ filter followed by a ‘Tube’ filter allows the centreline and radius information to be viewed as a series of tubes.

## Recent publications enabled by Chaste

Since our last publication on Chaste, over 70 peer-reviewed publications have been enabled in the areas described below.

Publications using Cardiac Chaste have included scientific studies relating to: basic mechanisms of cardiac electrophysiology and tissue structure in healthy and diseased settings; the sources and consequences of inter-subject electrophysiological variability; predicting the effects of drugs on cardiac activity, including safety assessment and the development of associated web-based tools (Cooper, Scharm, & Mirams, 2016). Other studies enabled by Cardiac Chaste have advanced technical methodologies for parameter identifiability and inference, model selection and uncertainty quantification in cardiac electrophysiology models (Johnstone et al., 2016); and for the verification and efficient numerical simulation of cardiac models (Green, Bohn, & Spiteri, 2019). The continuum-mechanics solvers in Chaste have been used for studies of cardiac electromechanics (Carapella et al., 2014).

The Cardiac Chaste code has also been applied to gastric electrophysiology, in particular focusing on the interstitial cells of Cajal network (Sathar, Trew, & Cheng, 2015).

Publications enabled by Cell-based Chaste have focused on: the cellular mechanisms and dynamics of intestinal homeostasis and carcinogenesis; the mechanisms underlying vascular tumour growth and response to therapy in the Microvessel Chaste project (Grogan et al., 2017); the biomechanical characterization of epithelial tissue development and wound healing; the organisation and proliferation of stem and pluripotent cells in development; the spread of sexually-transmitted infections; vascular remodelling (Osborne & Bernabeu, 2018); cell-based model calibration and parameterisation (Kursawe, Baker, & Fletcher, 2018); and their efficient numerical solution (Cooper, Baker, & Fletcher, 2017).

Papers on Lung Chaste describe its use for patient-specific airway tree generation and flow modelling (Bordas et al., 2015).

## Figures and Tables

**Figure 1:**
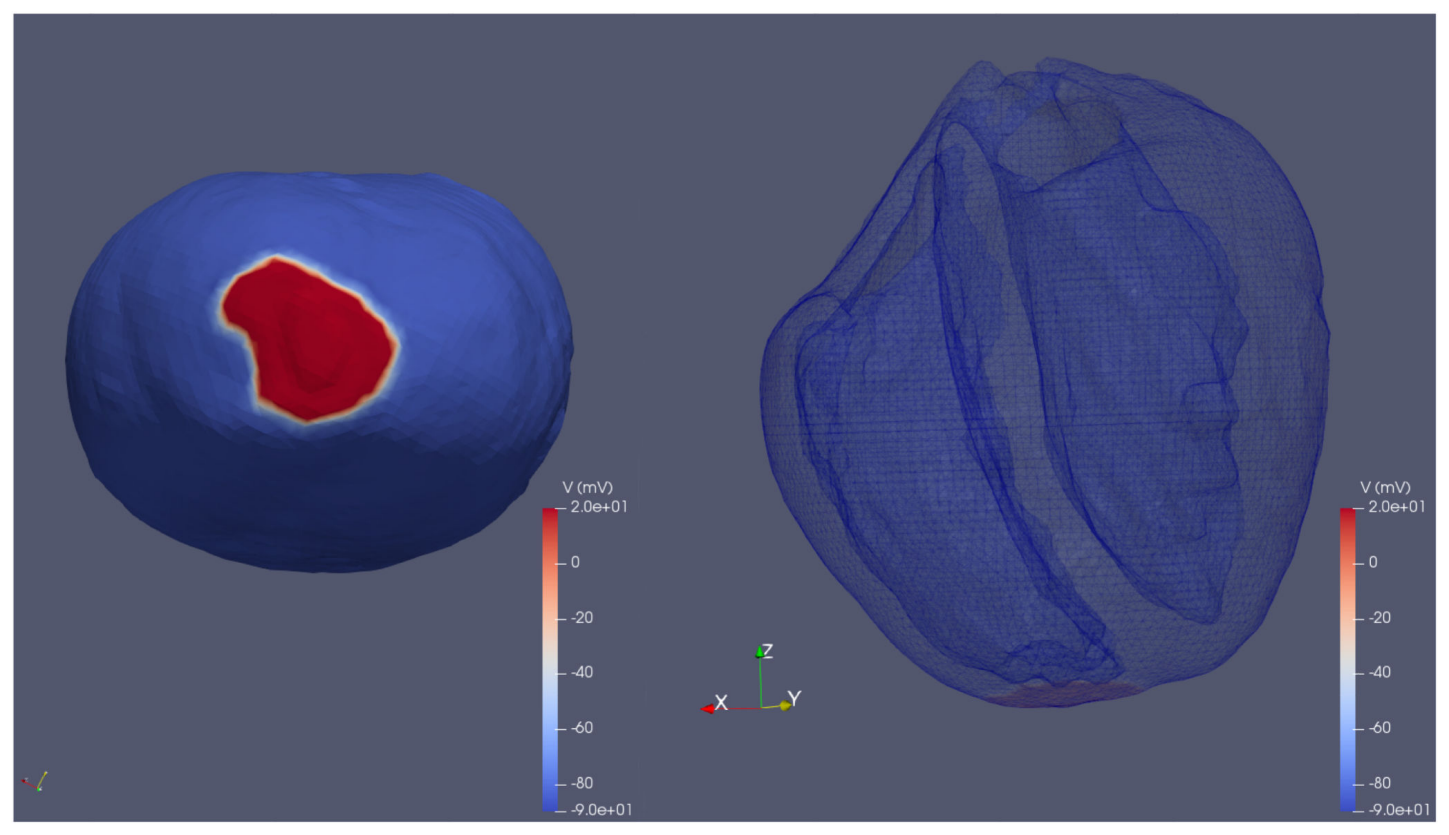
Trans-membrane voltage on the rabbit heart mesh at the end of the simulation. As viewed on the surface from the apex of the heart (left) and on a wireframe showing the ventricular cavities (right).

**Figure 2:**
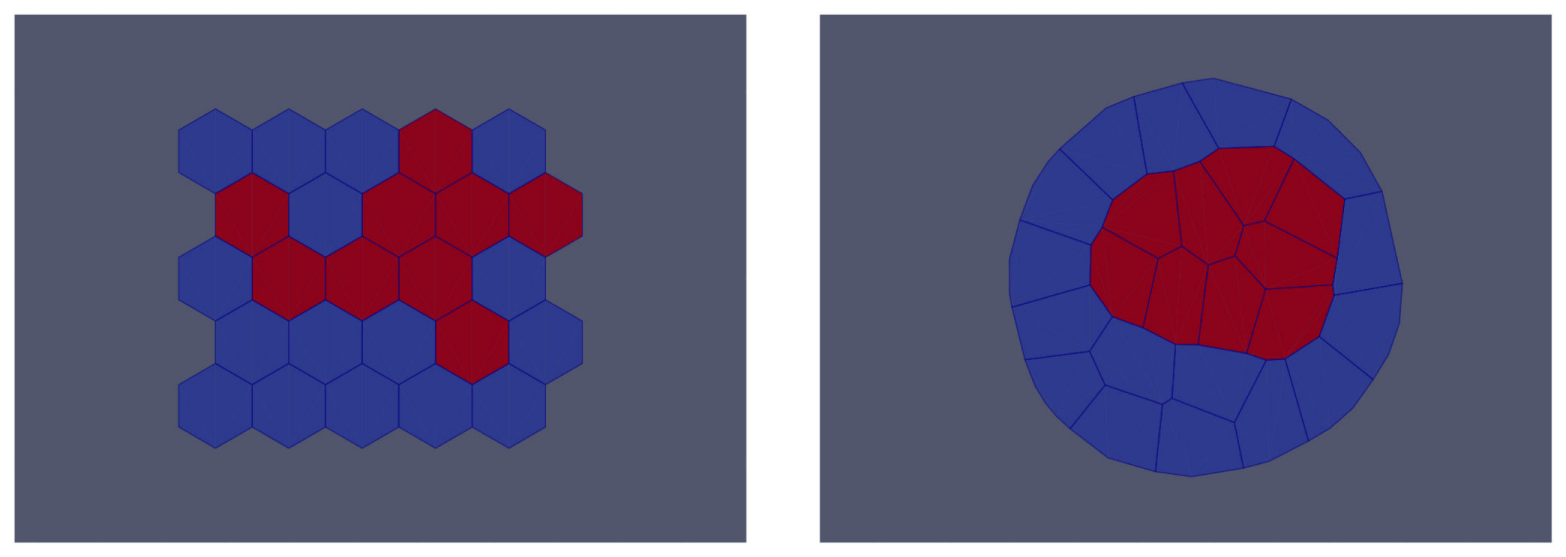
The initial configuration of cells (left), and the final configuration of cells after sorting has occurred (right).

**Figure 3:**
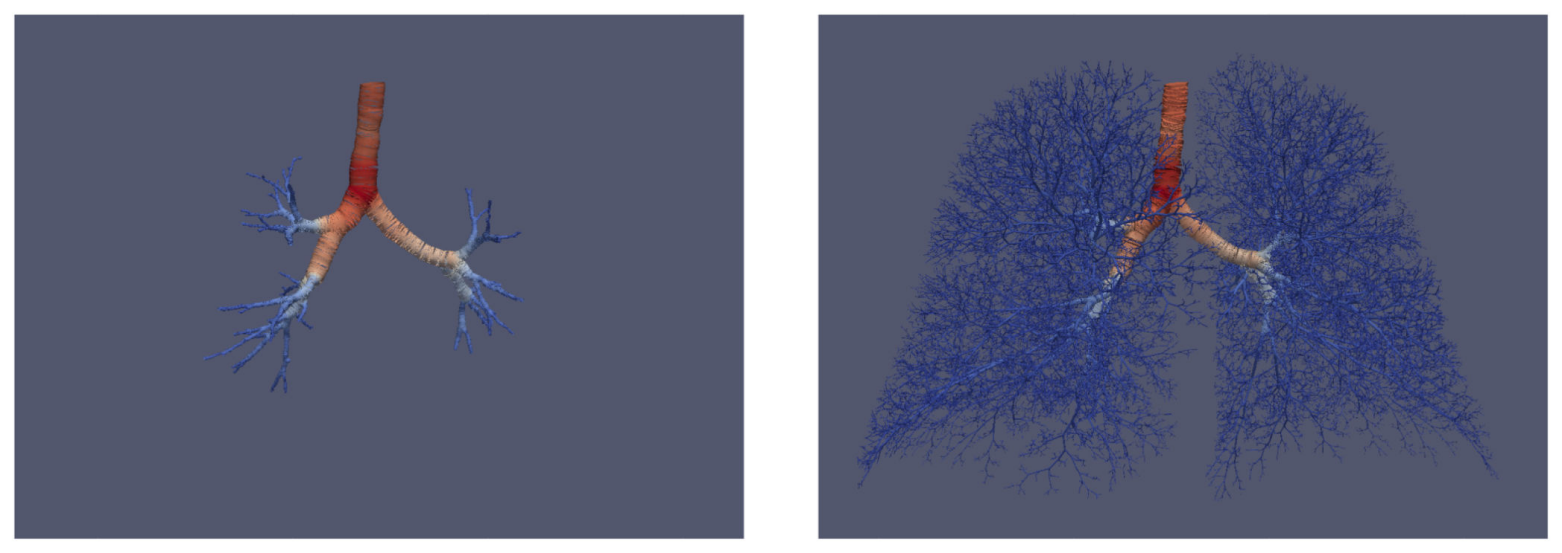
The initial geometry of major airways segmented from a CT scan (left), and an example of a complete generated airway tree (right).

**Table 1:** 

Software	Open Source	GUI	CA	CP	PM	VT	VM
Chaste		x	x	x	x	x	x
CompuCell3D	x	x		x			
Morpheus	x	x		x			
EPISIM		x			x		
CellSys		x			x		
PhysiCell	x				x		
Biocellion					x		
VirtualLeaf	x	x					x
EmbryoMaker	x	x			x		

A comparison of software tools for cell-based modelling. GUI: graphical user interface. CA: cellular automata. CP: cellular Potts. PM: particle model, a cell-centre model. VT: Voronoi tessellation, a cell-centre model. VM: vertex model. References: CompuCell3D (Swat et al., 2012), Morpheus (Starruß, Back, Brusch, & Deutsch, 2014), EPISIM (Sutterlin, Kolb, Dickhaus, Jager, & Grabe, 2013), CellSys (Hoehme & Drasdo, 2010), PhysiCell (Ghaffarizadeh, Heiland, Friedman, Mumenthaler, & Macklin, 2018), Biocellion (Kang, Kahan, McDermott, Flann, & Shmulevich, 2014), VirtualLeaf (Merks, Guravage, Inzé, & Beemster, 2011), EmbryoMaker (Marin-Riera, Brun-Usan, Zimm, Välikangas, & Salazar-Ciudad, 2015).
